# What Is the Significance of Placental Lakes in Pregnancy? A Historic Literature Review

**DOI:** 10.3390/jcm14041260

**Published:** 2025-02-14

**Authors:** Joanna Choi-Klier, Stephanie Masters, Danielle Lewis, Kaitlyn Taylor, Everett F. Magann

**Affiliations:** 1 Department of Obstetrics and Gynecology, Virginia Tech Carilion School of Medicine, Roanoke, VA 24016, USAsmmasters@carilionclinic.org (S.M.);; 2 Department of Obstetrics and Gynecology, University of Arkansas for Medical Sciences, Little Rock, AR 72205, USA; 3 Department of OB-GYN, University of Arkansas for Medical Sciences, 4301 W. Markham St. Slot #518, Little Rock, AR 72205, USA

**Keywords:** placental lakes, pregnancy, maternal outcomes, fetal outcomes, placental thickness, fetal growth restriction

## Abstract

**Background/Objectives:** The presence of placental lakes has been recognized on obstetric ultrasounds for many years, although their influence on pregnancy and perinatal outcomes remains uncertain. Most studies evaluating outcomes are small and many outcomes are conflicting. The question remains whether placental lakes affect pregnancy outcomes and, if so, how and under what circumstances? The purpose of this review was to determine the incidence, diagnosis, pathology, management, and pregnancy outcomes to determine the influence of an isolated lake versus the influence of a lake with the presence of other factors on pregnancy and perinatal outcomes. **Methods:** Electronic databases (PubMed, OVID, CINAHI, Embase, and Web of Science) were searched. The only limitation was the abstract/paper had to be in English. The search years were 1980–2023. The search terms included “placenta lake” AND “pregnancy outcomes”. **Results:** Of 323 abstracts identified, 26 full articles were selected as the basis of this review. A number of adverse outcomes have been reported with placenta lakes, including hypertensive disorders of pregnancy, fetal growth restriction, and intrauterine fetal demise. Other studies reported no adverse outcomes. A number of factors in addition to the placental lake, such as the size of the lake, number of lakes, and presence of a thick placenta, might increase the risk of adverse outcomes. **Conclusions**: Unfavorable pregnancy outcomes may be related to placental lakes, particularly if the lakes are multiple and large and the placenta is thick. Additional large studies are needed to determine if antenatal surveillance is helpful.

## 1. Introduction

One of the earliest reports of a placental lake diagnosed by ultrasound and confirmed by pathology of the placenta was a case report by Cooperberg et al. in 1979 [1]. A placental cystic structure observed on ultrasound was confirmed by pathology of the placenta to be a 3 cm × 4 cm × 3 cm cavity within the villous tissue. This case report was important as it highlighted the importance of correlating the image seen on ultrasound with the findings of pathologic examination of the placenta.

An early clinical study followed 40 patients with “subchorionic placental lucencies” measuring greater than 2 × 2 cm to determine if these pregnancies were complicated by adverse outcomes [2]. The investigators observed that unless the pregnancy was complicated by fetal anomalies or other intra-placental anomalies, these pregnancies were not at risk of unfavorable outcomes. A more recent study suggested an association between a thick placenta with placenta lakes and an increased risk of a poor pregnancy outcome [3].

Although the presence of placenta lakes has been documented for over 40 years, the clinical implications of finding a placental lake on an ultrasound examination is based on a limited number of studies, many of which are small. The purpose of this assessment is to review the literature and determine incidence, association, diagnosis, management, and impact on perinatal outcomes of placental lakes identified by ultrasound in pregnancy.

## 2. Methods

**Figure 1:**
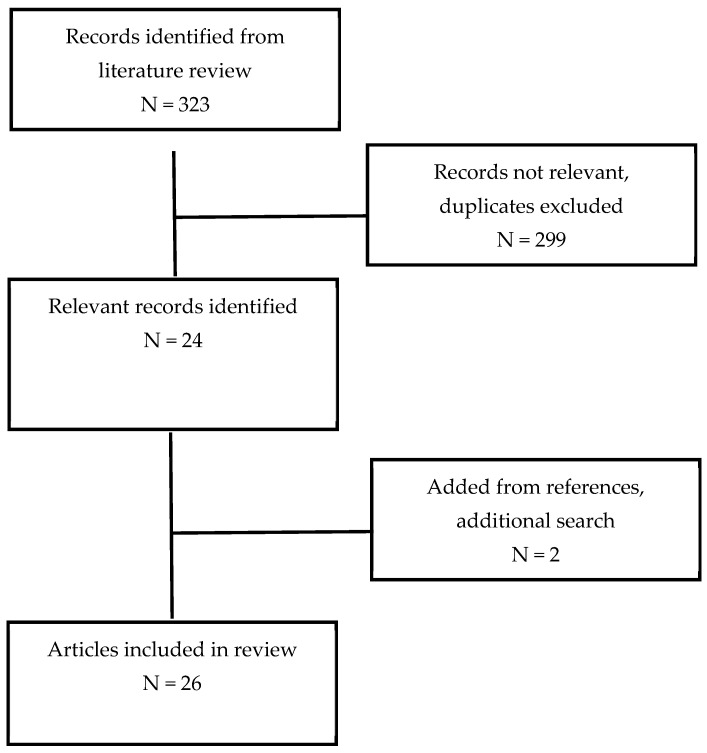
Literature review.

A literature search was carried out by a university librarian at the University of Arkansas for Medical Sciences (UAMS). The search engines used were PUBMED, OVID, CINAHL, Embase, and Web of Science. The search terms used were “placental lake” OR “placental lakes” OR “pools of blood” AND “pregnancy outcomes” OR “etiology” OR “risk factors” OR “imaging” OR “diagnostic imaging” OR “pathology” OR “physiology” OR “preeclampsia” OR “miscarriage” OR “stillbirth” OR “preterm birth”. The search was limited to the English language, and the years searched were 1980 to 1 August 2023. The authors independently reviewed the literature search results for relevant abstracts. There were a total of 323 abstracts identified. The full articles of the abstracts that were relevant to placenta lakes and pregnancy outcomes were obtained and read. After removing duplicate papers, we determined that 24 articles contained applicable information for this review. The Investigational Review Board of the University of Arkansas for the Medical Sciences determined that since this review is of previously published articles, it does not fall under human subject research oversight. The references of these full articles were examined for any additional relevant articles. A final total of 26 articles were identified that became the basis of this review (Table 1) (Figure 1).

### 2.1. Pathology

**Figure 2:**
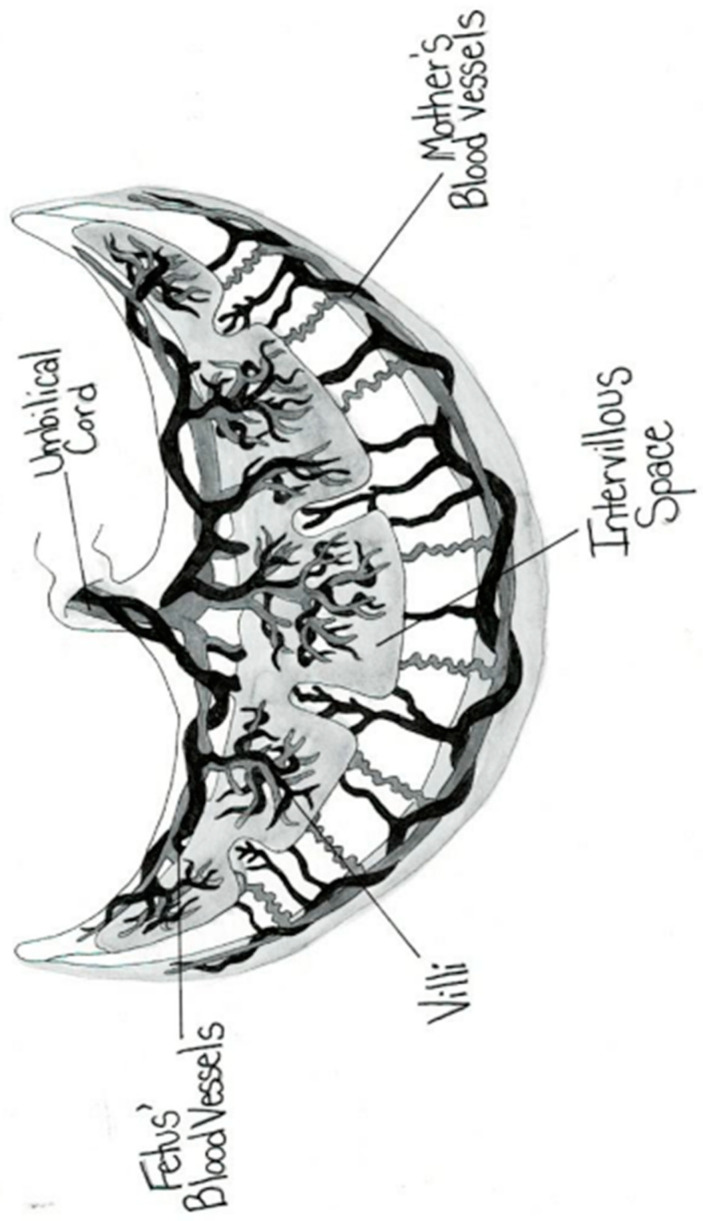
Normal placental.

**Figure 3:**
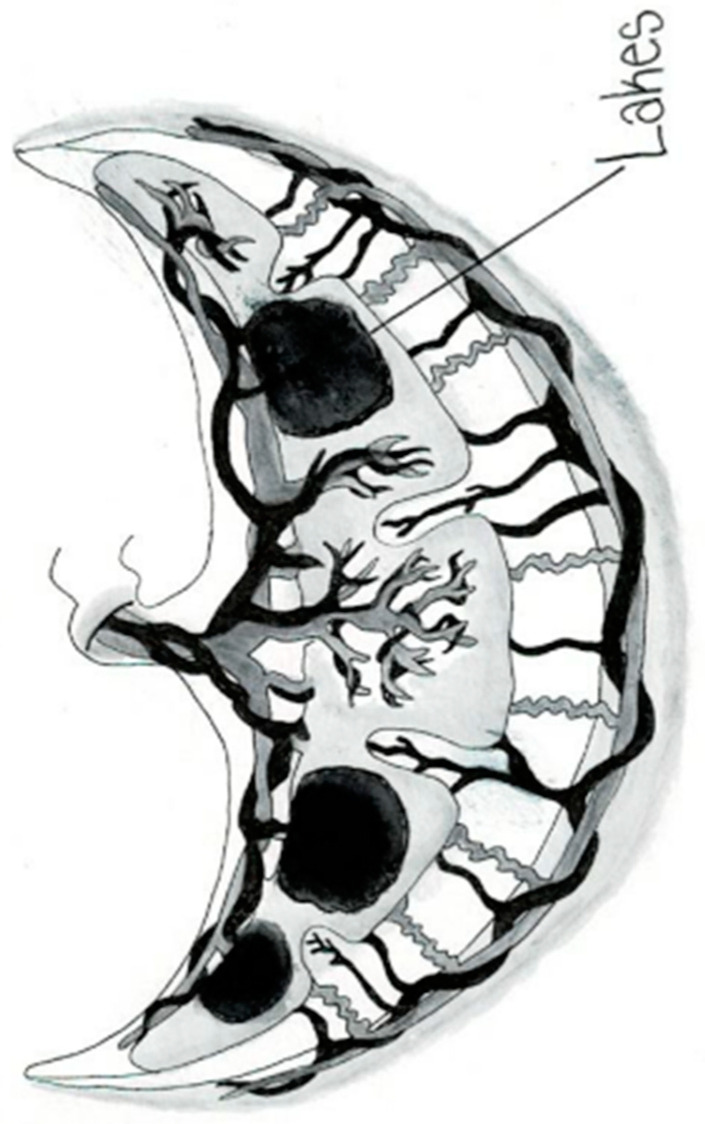
Placental lakes.

**Figure 4:**
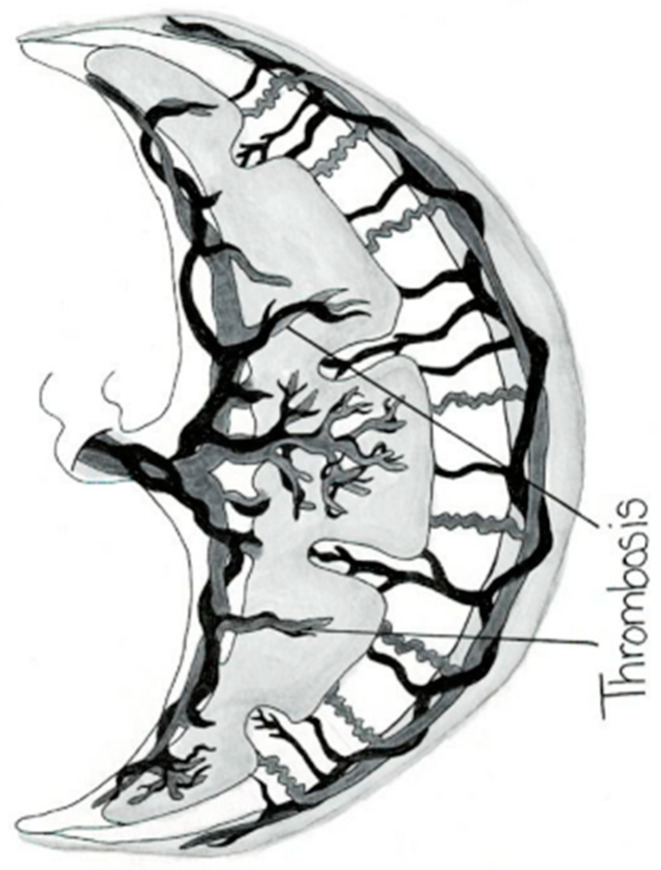
Placental intervillous thrombosis.

The placental circulation in pregnancy occurs through two independent circulation systems in which the two systems do not mix: (1) fetoplacental and (2) uterine/spiral artery/placenta. In the uterine/spiral artery system, the maternal arterial blood flows into the spaces surrounded by the anchoring villi and leaves these spaces by the uterine veins [13] Figure 2. A placental lake, also labeled as a placenta cavern, is defined as a homogeneous, sonolucent (allowing the production of waves without echoes), avillous, vascular space containing only maternal blood and is surrounded by the normal placental parenchyma (Figure 3). Swirling maternal blood can be demonstrated by either grayscale imaging or low-scale color Doppler flow studies. Intervillous thrombosis can occur in placental lakes, with clotting of the maternal blood and the combination of placental lakes and intervillous thrombosis often seen together [25]. The prevalence of an intervillous thrombi is increased in pregnancies with fetal growth restriction and in pregnancies complicated by diabetes [25,26]. The intervillous thrombus shows no swirling blood flow and is more likely to be hypoechoic (area appears dark gray) because not as many echoes are returned as opposed to sonolucent areas, which appear black, with no echoes demonstrated on ultrasound (Table 2) (Figure 4).

The appearance of placental lakes on ultrasound is brought about by the low echogenicity of villous-free areas within the placenta, filled with maternal blood of varying velocities [25].

### 2.2. What Are Placenta Lakes vs. What Are Placenta Lacunae?

**Figure 5:**
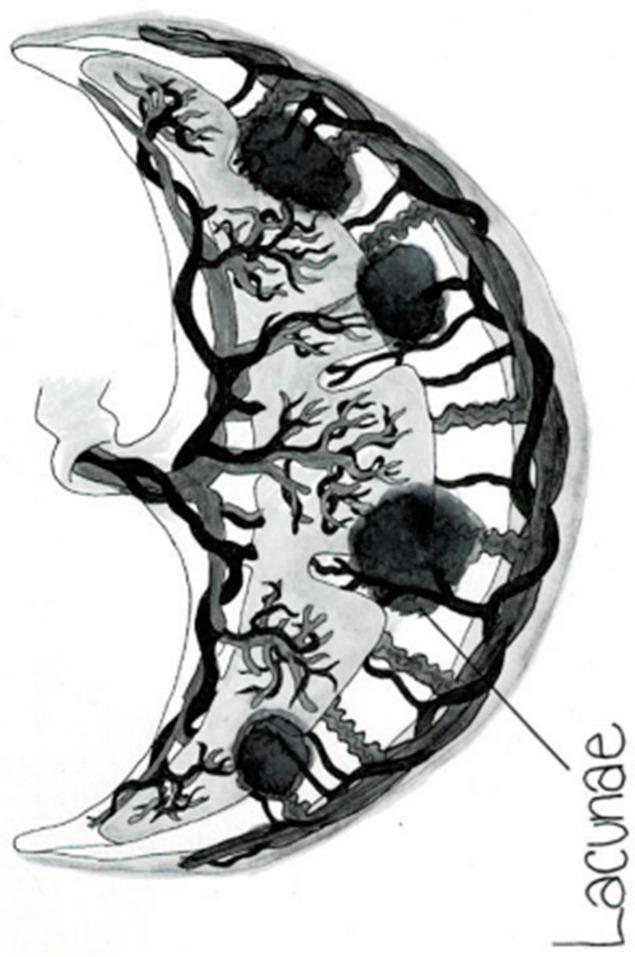
Placental lacunae.

Anechoic placental zones have earned numerous names, including “placental lakes”, “placental venous lakes”, “placental lacunae” and “placental caverns” [9]. Both placental lakes and lacunae are sonographic sonolucent spaces without villi and in close proximity to normal placental tissue (Figure 3 and Figure 5). Placenta lakes are commonly recognized as homogenous anechoic areas surrounded by normal placental tissue at the time of the targeted ultrasound but can also be seen near the end of the first trimester as maternal blood starts freely flowing into the intervillous spaces. Lakes are more frequently seen under the fetal plate and at the placental periphery and in the center of a lobule where there is less villous tissue [12] (Figure 3).

Lacunae are seen to be developing during the second trimester. They represent the distortion of placental lobules that develop within a uterine scar as the result of high-volume, high-velocity blood flow [24]. The placental lacunae are defined as large, irregular sonolucent spaces that give the placenta a “moth-eaten” appearance on ultrasound grayscale imaging. (Figure 5) The presence of placental lacunae later in pregnancy has become synonymous with a diagnosis of placenta accreta spectrum (PAS) and there is an association between the number of lacunae and the severity of PAS. Jauniaux et al. examined 11 standardized ultrasound signs associated with a high probability of PAS at birth and found that out of those 11 standardized signs, “placental lacunae” is the only significant ultrasonographic sign [12]. The distinction between lakes and lacunae becomes somewhat confusing as different authors appear to interchange lakes with lacunae (Table 3). Some examples are in the findings of a prospective cohort study [24], where it was stated that the ultrasound findings of multiple placental lakes are linked with placenta accreta in patients who also have a cesarean scar.

Lakes and lacunae can co-exist within the placenta and both will evolve in size and shape as the pregnancy progresses. Lakes will appear toward the end of the first trimester; however, lacunae, seen in the accreta spectrum, will develop progressively during the second trimester, with color Doppler showing high-volume, high-velocity flow from the radial and arcuate arteries. The difference between lakes and lacunae is their connection with changes in the underlying circulation and the scaring from prior uterine surgery, as is seen with lacunae [12]. This study examines the associations between placental lakes and pregnancy outcomes.

### 2.3. Prevalence

Jauniaux et al. (2024) reported that the prevalence of placental lakes in the general population varies widely from 2 to 71% due to the varying use of different definitions to define placental lakes and the wide range of gestational ages at the time of the ultrasound examination [12].

### 2.4. Histologic Findings with Placental Lakes

Jauniaux et al., in uncomplicated pregnancies, observed that the presence of placental lakes on ultrasound was not associated with changes in the surrounding villous structure on postpartum histopathologic assessment and the pathologic findings are not related to the size of the lake [12]. By contrast, pregnancies complicated by vasculopathies and malperfusion of the placenta frequently present with a combination of secondary placental macroscopic lesions, including intervillous and parabasal thrombosis, hematomas, infarctions, and extensive fibrin deposition, on histopathologic exam. This may identify another more complex type of placental lake.

A case by Muramatsu et al. reported a hypoechoic lesion measuring 2–4 cm in the retroplacental area that was delivered by cesarean. Because of the potential risk of hemorrhage, an elective cesarean section was performed at term [21]. Although no abnormal findings were noted at the maternal–placental interface, histologic exam showed chorangiosis (an abundance of blood vessels in the placental villi) in the terminal villi. Soma et al. reported cases of large placental lakes and chorangiosis in the placenta among residents of the Himalayas, suggesting that chronic maternal hypoxia of individuals living at high elevations may lead to hypervascularization of the placenta [23]. The histologic findings of this case may suggest unidentified disturbances in maternal–fetal oxygen exchanges, which are associated with the large placental lake.

### 2.5. Diagnosis/Imaging

Placental lakes are defined as homogenous anechoic areas of >2 cm in diameter, surrounded by placental tissue of normal echogenicity, and are the most common placental finding on greyscale ultrasound imaging [12]. They are usually identified at the time of the fetal anatomic survey. Placental lakes can sometimes appear like the early stages of thrombosis on ultrasonography. Morikawa et al. described two case reports utilizing MRI to differentiate between these two diagnoses. On MRI, the lakes showed low isointensities on T1- and T2-weighted MRI, suggesting fresh blood, as opposed to high isointensities on T1- and T2-weighted MRI, which would represent a thrombosis [15].

Alternative imaging sources have been tried to improve the classification of placental lakes. Inubashiri et al., in a 2014 case report, used three-dimensional high-definition flow (3D-HDF) to diagnose a placental lake [10]. The investigators reported that a sonolucent area in the placenta was found on 2D ultrasound (US) and 3D ultrasonography, but the 3D-HDF US clearly showed the 3D structure of the lesion, with a cavity and possible blood pooling inside the structure, and concluded that 3D-HDF could help with the investigation of abnormalities relevant to perinatal outcomes [10].

**Figure 6:**
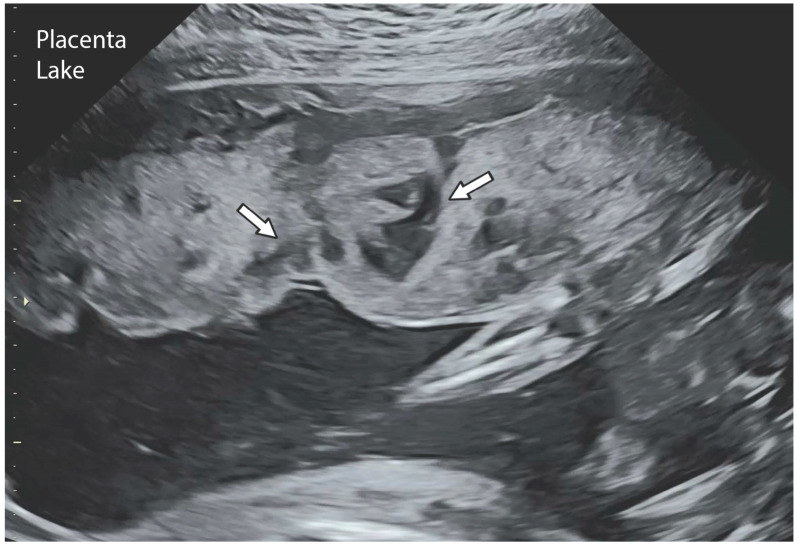
Placental lake on ultrasound. The arrows indicate placental lakes on ultrasound.

**Figure 7:**
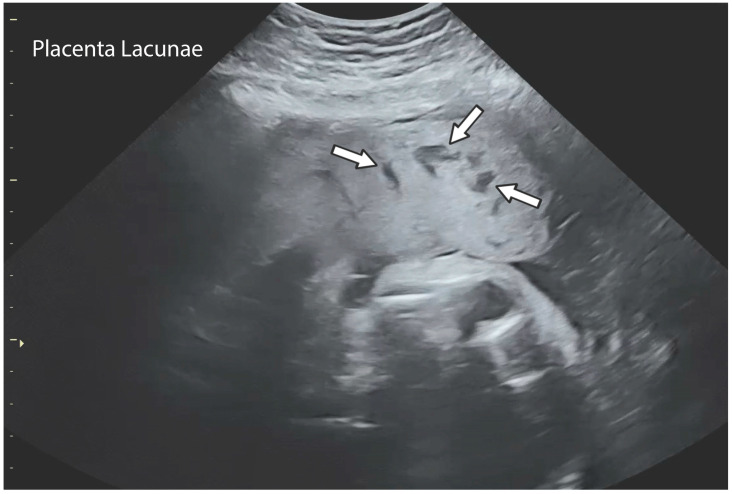
Placental lacunae on ultrasound. The arrows indicate placental lacunae on ultrasound.

Inubashiri et al. also used spatiotemporal image correlation (STIC) with high-definition flow (STIC-HDF) to evaluate the angiographic vascular flow of placental vessels. In this case study, the ultrasound examination showed asymmetrical fetal growth restriction (FGR) with a 2-week smaller abdominal circumference and a placental lake. They used STIC-HDF to depict the actual conditions of a large spiral artery jet feeding into the placental lake with turbulence. At delivery, the examination of the placenta showed subchorionic spaces surrounded by normal villi and fibrin deposition without subchorionic hematoma or chorionic angioma, consistent with a diagnosis of a placental lake [11] (Figure 6 and Figure 7).

### 2.6. Clinical Management

Controversy remains over whether placental lakes require surveillance and alterations in delivery planning due to conflicting results on the clinical significance of placental lakes. The route of delivery in a pregnancy with a placental lake is typically determined by the usual obstetric indications rather than because of the presence of the placental lake(s).

Two case reports of a large placental lake measuring (114 × 48 × 40 mm) in the first case and (66 × 37 × 22 mm) in the second case were both diagnosed by ultrasound and MRI and confirmed to have turbulent blood flow [15]. Because of the size of the lake in the first case, the fetal well-being was closely monitored during the pregnancy. At 37 weeks, because of concern that the lake might rupture during delivery, the fetus was delivered by elective cesarean. The neonate was small for the gestational age and weighed 1940 g. The child’s health was followed, and at the time of this report, the child was 2 years old with normal growth and development. The second case had close monitoring of fetal well-being and was delivered by an elective cesarean at 37 weeks. The neonate was small for the gestation age, weighing 2195 g. The child, at the time of the report, was 5 months old, with normal growth and development. An assessment of the fluid within the lake was only possible in the second case and was consistent with maternal blood [15]. Has et al. described the case of a placenta lake under the membranes between the two lobes of a bilobed placenta with a cord insertion site through the membranes, with the vessels extending to each lobe of the placenta [14]. The lake was large, measuring (10 × 10.3 × 10 mm), with observed blood flow turbulence. This was a referred patient, and she was having contractions when seen. At the time of the contractions, the placental lake decreased in size, and after the contraction, returned to its normal size. At the end of the 36th week of pregnancy, the patient went into labor, with an uneventful first stage of labor. In the second stage, with delivery of the head, there was a sudden gush of blood, with the delivery of a 3100 g fetus delivered with Apgar scores of 9 and 10 at 1 and 5 min, respectively. The mother had estimated blood loss of 1500 mL, but the bleeding quickly responded to oxytocin and misoprostol [14]. A third case was a 32-year-old woman with a history of maternal cardiac disease and a large placental lake seen measuring (100 × 65 × 50 mm) on ultrasound. The patient was on heparin during her pregnancy because of her complex cardiac condition. She had massive vaginal bleeding at 20 weeks and the heparin was stopped and patient was continued on aspirin. At 38 weeks, the patient was delivered by elective cesarean due to her maternal cardiac condition. The fetus weighed 2375 g and had normal Apgar scores [16]. It may be reasonable in rare cases to offer elective cesarean section based on the size and location of the placental lakes; however, there is not enough data to prove improved safety over vaginal delivery. Therefore, shared decision making may be appropriate. Based on current data, there is no indication for early delivery based solely on the presence of placental lakes.

### 2.7. Perinatal Outcomes

Due to contradictory results in the current literature, there is doubt about whether placental lakes affect perinatal outcomes.

Cooley et al., in a retrospective study, evaluated the influence of the placental architecture on pregnancy and perinatal outcomes. The outcomes evaluated were the primarily outcomes of uteroplacental perfusion (preeclampsia, small for gestational age and fetal growth restriction). The placental architecture assessed included the placental implantation site and cord insertion site into the placenta, the presence of placental thickness, placental calcifications and placental lakes. The only outcome associated with the presence of placental lakes was vaginal bleeding in the first trimester of pregnancy. There was no comment in the results or discussion that the first trimester bleeding resulted in increased first or second trimester pregnancy loss [8].

One such investigated outcome is a non-reassuring fetal status. Both Bien et al. and Hwang evaluated the significance of the placental lake size in relation to pregnancy outcomes. Bien at al. conducted a retrospective observational study of 1728 women, of whom 156 had placental lakes [7]. The placental lakes were divided into small lakes (<20 cm^3^ n = 129), medium lakes (≥20 cm^3^ and <30 cm^3^; n = 10) and large lakes (>30 cm^3^; n = 17). They observed that women with large lakes, even after an adjustment for potential confounders, had a significantly increased risk of a non-reassuring fetal status (OR = 7.26; 95% CI = 1.53–29.84, *p* for trend = 0.005). A non-reassuring fetal status was defined in their study as the persistent worsening of a Category II pattern or Category III pattern [7]. Hwang et al., in 2012, performed a prospective observational study of 113 placental lakes to determine if there was an association between the size of the placental lake and an adverse pregnancy outcome [4]. This was a prospective observational study of 1294 singleton pregnancies without fetal malformations and chromosomal abnormalities between 19 + 0 weeks and 23 + 6 weeks. Placental lakes were defined as a lake when their measurement was greater than 2 cm × 2 cm in diameter with turbulent flow. There were 113 pregnancies that met this criterion. Placenta lakes > 5 cm in their maximum lengths were defined as large lakes. These large lakes were correlated with small-for-gestational-age fetuses. The investigators recommend a second trimester growth scan and antenatal surveillance if growth restriction is found [4]. Both of these studies support the idea that while not all placental lakes are clinically significant, larger lakes may hold relevancy to pregnancy outcomes.

While there are studies that found significant differences in the outcomes of pregnancies with placental lakes, there are several studies that demonstrated no significant difference in outcomes. A prospective observational study by Reis et al. challenged the idea that placental lakes increase the risk of small-for-gestational-age neonates and other adverse outcomes [5]. In this prospective observational study, the placentas of 4106 women who were without maternal or fetal disease were examined between 15 and 34 weeks. Full records and outcomes were available on 59/92 pregnancies with lakes defined as > 2 cm. This group was compared to a control group of 37 pregnancies without maternal or fetal disease and a normal placenta. There were no differences in the adverse pregnancy outcomes, defined as the number of stillbirths or neonatal deaths, small for gestational age, amniotic fluid volume and preterm delivery, between the study and control groups [7].

Mansoor et al. conducted a retrospective cohort study to assess the clinical significance of placental lakes [22]. This was a retrospective study that evaluated if there is an association with the presence of or size of placenta lakes and adverse pregnancy outcomes. The adverse pregnancy outcomes evaluated included low birth weight, cesarean delivery, primary cesarean for non-reassuring fetal heart tracing, fetal growth restriction, preterm birth and severe preeclampsia. A total of 1052 patients were evaluated, of whom 294 had placental lakes. There were 204 small placentas (>2 cm and <4 cm) and 90 large placentas ≥ 4 cm. Neither the presence nor the size of the placental lakes was associated with adverse pregnancy outcomes [22]. This challenges previous studies’ findings that larger placental lakes hold clinical significance.

Schifer et al. performed a systematic review of healthy placentas compared with unhealthy placentas, which were labeled as placenta syndrome pregnancies [17]. Placenta syndrome pregnancies were pregnancies with preeclampsia, pregnancy-induced hypertension, and fetal growth restriction. An aging placenta (development of placental aging syndrome) was identified by increasing placental thickness, placental lakes, and placental calcifications. For the purpose of their analysis, they used increasing placenta thickness as a continuous variable and the presence of placental lakes or calcifications as prevalence. Placental lakes were identified in five of the studies, but the definitions of a placental lake varied among the studies. The hypothesis of the study was that lakes were associated with preeclampsia and fetal growth restriction. In this systematic review, there was no statistical association found between placental lakes in the women with healthy and unhealthy pregnancies [17].

Thompson et al. looked at whether an association exists between the finding of placental lakes at the 20-week scan and an increased risk of uteroplacental complications or a poor pregnancy outcome [6]. They found no correlation between the presence of placental lakes and a small-for-gestational-age infant below the 5th or the 10th centile for birth weight, or no association with the development of preeclampsia, placental abruption, perinatal mortality, or the overall occurrence of “any pregnancy complications”. They did observe that a placental thickness greater than 3 cm at 20 weeks gestation was significantly associated with the presence of placental lakes on ultrasound and that a combination of increased placental thickness and lakes was associated with an increased risk of severe (preterm) preeclampsia [6]. Wan Masliza et al., in a prospective study of placental lakes and increased placental thickness, observed a 3.4 times higher risk of developing hypertensive disease during pregnancy, although the results were not statistically significant [3].

Some case reports suggest a link between adverse pregnancy outcomes and uteroplacental complications. Bursac et al. described a case where multiple lakes seen in early pregnancy were associated with decreased umbilical artery blood flow and fetal growth restriction [18]. Kawakita et al. reported a case of severe fetal growth restriction associated with a subchorionic placental lake and reverse end-diastolic flow on Doppler flow studies resulting in a cesarean delivery at 27 weeks [19]. Jauniaux et al. reported a case of fetal growth restriction in a patient who had multiple placental lakes within the placental tissue [20]. This patient underwent medical termination due to a follow-up ultrasound showing no fetal growth and poor prognosis. Histopathological exam showed several intervillous thromboses with villous necrosis involving half of the placental tissue [20]. Although placental lakes are considered benign, cases such as these involving early second trimester fetal death highlight that lakes may affect fetal growth through disturbances or redistribution of the fetoplacental circulation and that these are not clinically meaningless since placental abnormalities can have implications for antepartum surveillance or therapy (Table 4).

## 3. Discussion

This review analyzed studies on placental lakes, reporting findings related to their incidence, imaging, pathology, clinical management, and association with pregnancy outcomes. This review revealed significant variability in the reported prevalence of placental lakes (2–71%), attributable to the inconsistent definitions and imaging methodologies. Histopathologically, placental lakes were generally not associated with structural changes in the surrounding villous tissue in uncomplicated pregnancies but were linked to secondary placental lesions (e.g., thrombosis and fibrin deposition) in pregnancies with vasculopathies or placental malperfusion.

The findings of this review highlight both the potential significance and the current uncertainty surrounding placental lakes in clinical practice. While small and isolated placental lakes appear to be benign in most pregnancies, larger or multiple lakes, especially when accompanied by increased placental thickness, may warrant closer monitoring. These cases have been associated with adverse outcomes, including FGR and hypertensive disorders, suggesting potential mechanisms related to abnormal placental vascularization, localized hypoperfusion, or placental insufficiency.

For clinicians, these results emphasize the importance of precise characterization and documentation of placental lakes during routine ultrasound evaluations. Differentiating between benign placental lakes and lacunae associated with placenta accreta spectrum is critical, as misclassification could lead to unnecessary interventions or missed diagnoses. Advanced imaging techniques, such as 3D Doppler and spatiotemporal image correlation (STIC), may aid in improving diagnostic accuracy. Professional organizations might consider advocating for standardized criteria for the detection and reporting of placental lakes, which could reduce the variability in interpretation and improve the comparability across studies. Uniform guidelines would also aid clinicians in determining when additional monitoring or intervention is warranted.

The clinical management recommendations vary. There is little to no research exploring the utility of and approach to antenatal surveillance and management of placental lakes and its effect on pregnancy outcomes. While some studies suggest that placental lakes demonstrate no significance in terms of the influence of perinatal outcomes, some studies do find associations with non-reassuring fetal status, FGR/SGA, and preeclampsia. This may suggest that placental lakes are in some cases a significant finding, although it still remains unclear when significance is met. Based on the available research, this may be when placental lakes are numerous, large (>4–5 cm), or associated with placental thickening. Given the current data, it is reasonable to offer serial fetal growth assessment and antenatal surveillance (NST vs. BPP) in the third trimester to those patients with multiple lakes, large lakes, or lakes associated with placental thickening, although more research needs to be performed in order to establish that this is beneficial and necessary. Elective cesarean delivery was occasionally chosen for large placental lakes to mitigate concerns over potential hemorrhage, although no significant increase in postpartum hemorrhage was observed. These findings underscore the need for standardization in diagnostic criteria, larger cohort studies, and prospective research to clarify the clinical significance of placental lakes.

Given the inconsistent evidence linking placental lakes to adverse pregnancy outcomes, further research is required before these findings can be confidently applied in clinical settings. Future studies should focus on prospective, large-scale cohort designs with standardized imaging protocols and clear definitions of placental lakes. Additionally, research into the underlying pathophysiology of placental lakes and their potential impact on placental function could elucidate the mechanisms driving adverse outcomes and inform targeted interventions. Until such data are available, clinicians should tailor their approach to individual patient risk profiles.

The strengths of this review include that it provides a comprehensive synthesis of the existing literature, including studies with diverse methodologies and populations, which enhances its generalizability. Additionally, this analysis highlights both positive and negative findings, ensuring balanced interpretation and avoiding overgeneralization. The limitations include the variability in definitions and diagnostic criteria for placental lakes across studies, which introduces potential bias and reduces comparability. Additionally, most included studies were retrospective or observational, limiting the ability to infer causality. Also, the small sample sizes in many studies, particularly for adverse outcomes, reduce the generalizability. These limitations, however, underscore the need for future research employing standardized protocols and robust study designs to build on the insights provided by this review.

In conclusion, this review highlights the nuanced role of placental lakes in pregnancy, with small and isolated lakes generally considered benign while larger or numerous lakes may signal an increased risk of adverse outcomes. However, the variability in diagnostic criteria and inconsistent findings limit the current utility of placental lakes as prognostic markers.

## Figures and Tables

**Table 1:** Placental Lakes Articles Used in this Review.

Author	Publication Year	Country	GA at US	Number of Patients	Placental Lake Size (cm)	Outcome
Hwang [4]	2012	Korea	19–23 + 6	1294 (109 with lakes)	2 × 2, large > 5 in length	Large placental lakes are correlated with small-for-gestational-age status
Reis [5]	2005	Brazil	15–40	4106 (92 with lakes)	>2 × 1	No diff in birth wt, PTD, adverse outcomes
Thompson [6]	2002	UK	18–22	1198 (213 w/lakes)	2 × 2	No diff in IUGR, PIH, severe PE or abrupt
Bian [7]	2022	China	20–24	1728 (156 w/lakes)	1	No diff in NR-FHR, higher vol lakes had increased NR-FHR
Cooley [8]	2011	Ireland	2nd and 3rd trimester	810	Not stated	Placental lakes more common in women with first trimester bleeding
Gavanier [9]	2021	France	All trimesters	Not stated	>/=1 (lacunae), thickness > 4	Placental lakes or vesicles can signal a range of diseases and complications that are potentially serious for the mother or the fetus
Inubashari [10]	2001	Japan	36 + 3	One	Not stated	Using spatiotemporal image correlation with high-definition flow (STIC-HDF) to evaluate placenta lake
Inubashari [11]	2001	Japan	16	One	3.0 × 2.8 × 4.0	Applying three-dimensional high-definition flow (3D-HDF) for diagnosing placental lakes
Jauniaux [12]	2023	UK	20–23 weeks	Ten	Homogenous anechoic areas of >2 cm in diameter	Placental lakes, including large ones, have no clinical significance; placental lacunae develop progressively during the second trimester in pregnancies complicated by PAS at birth
Cooperberg [1]	1979	Canada	32	One (case study)	3 × 4 × 5	One of the earliest reports of a placental maternal lake diagnosed by ultrasound and confirmed by pathologic examination
Katz [2]	1991	US	19.3 weeks (mean)—range 13 to 34	46	>2 × 2	Subchorionic placental lucencies do not appear to be associated with adverse pregnancy outcomes
Yoshihara [13]	2022	Japan	Not relevant	Not relevant	Not relevant	The placental circulation in pregnancy occurs through two independent circulation systems in which the circulation systems do not mix
Wan Masliza Wan Daud [3]	2005	Malaysia	20–22 w and 30–32 w	68	Presence of small placental lakes more than 3, or less than 3 but large (more than 2 cm in diameter)	Presence of abnormal placental thickness and lakes at 30–32-week scan is associated with maternal hypertensive disease, fetal growth restriction and low umbilical cord pH; however, these were not statistically significant
Has [14]	2001	Turkey	28 w	1	10 × 10.3 × 10	No complications during pregnancy; however, obstetric hemorrhage took place during second stage of delivery
Morikawa [15]	2005	Japan	24 w and 35 w	2	11 × 4.8 × 4.0 cm; 6.6 × 3.7 × 2.2 cm	SGA infants were born at term in both cases; however, placental lakes considered benign finding
Di Donato [16]	2016	Italy	20 w	1	10 × 6.5 × 5	Lake resolved in the 3rd trimester, pt delivered via elective cesarean section due to maternal cardiac history and did not have a PPH
Schiffer [17]	2021	Netherlands	Between 12 and 31 weeks—for placental lakes specifically	28 studies were included, describing a total of 1719 cases of placenta syndrome pregnancies. For placental lakes specifically, a total of five studies	Generally, >2 × 2 cm surrounded by placental tissue of normal echogenicity	No statistically significant difference in presence of placental lakes between complicated and uncomplicated pregnancies
Bursac [18]	2014	Croatia	33 w	1	3.6 × 2.3	Incidental finding of a placental lake associated with several other risk factors for adverse outcome in high-risk pregnancy, resulted in premature birth and newborn pathologic brain US with clinical correlate
Kawakita [19]	2013	Japan	26 w	1	14 × 2.4 × 2.7 cm	Severe FGR associated with huge subchorionic placental lake and reversed MCA end-diastolic flow, resulted in a C-section at 27 w 3 d
Jauniaux [20]	1996	UK	12 w	1	Not stated	FGR in a patient who had multiple placental lakes who subsequently underwent medical termination as follow-up US showed no fetal growth and poor prognosis
Muramatsu [21]	2010	Japan	35 w	1	2–4 cm thick	Delivered by elective cesarean at term because of the potential risk of hemorrhage and lack of guidelines
Mansoor [22]	2022	US	20 w	210	Small < 5 cm; Large </= 5 cm	No difference in gestational age at delivery, preterm birth, C-section, preeclampsia or birth weight between groups with small vs. large placental lakes
Soma [23]	2005	Japan	All trimesters	6500	Not stated	Chorangiosis associated with placental lakes
Japaraj [24]	2007	Malaysia	>28 w	21	Not stated	Multiple placental lakes most common ultrasound finding in patients with a history of previous cesarean section diagnosed with placenta accreta

**Table 2:** Ultrasound imaging definitions.

Ultrasound Description	Definition
Sonolucent	Appears black on ultrasound (waves without echo)
Anechoic	No echo on ultrasound
Hypoechoic	Not many echoes on ultrasound and appears gray on ultrasound
Hyperechoic	Produces echoes of larger amplitude or density than surrounding tissue

**Table 3:** Placental lakes vs. placental lacunae.

Characteristics	Placental Lake	Placental Lacunae
Sonographic appearance	Anechoic area surrounded by normal tissue	Anechoic area surrounded by normal tissue
First ultrasound appearance	First trimester of pregnancy	Second trimester
Location	Areas of low villous density, under fetal plate, marginal areas	Distortion of placental lobular development around the prior uterine scar
Blood flow	Low volume or varying velocity	High volume and high velocity

**Table 4:** Proposed management of placental lakes.

	Fetal Growth Assessment	Antenatal Surveillance	Delivery Timing/Route
Isolated small (<4 cm) placental lakes	Third trimester growth with reassessment of placenta	Not recommended	Usual OB indications
Multiple placental lakes	Serial growth ultrasounds q3–4 weeks with continued placental evaluation	Consider weekly NST or BPP	Usual OB indications though shared decision making on route in certain cases is reasonable
Large placental lakes (≥4 cm)			
Thickened placental with lakes			
